# Stoichiometry of carbon, nitrogen and phosphorus is closely linked to trophic modes in orchids

**DOI:** 10.1186/s12870-023-04436-z

**Published:** 2023-09-12

**Authors:** Julita Minasiewicz, Adrian Zwolicki, Tomáš Figura, Alžběta Novotná, Melissa F. Bocayuva, Jana Jersáková, Marc-André Selosse

**Affiliations:** 1 Faculty of Biology, Department of Plant Taxonomy and Nature Conservation, University of Gdańsk, ul. Wita Stwosza 59, Gdańsk, 80-308 Poland; 2 Faculty of Biology, Department of Vertebrate Ecology and Zoology, University of Gdańsk, ul. Wita Stwosza 59, Gdańsk, 80-308 Poland; 3 Department of Mycorrhizal Symbioses, Institute of Botany, Czech Academy of Sciences, Lesní 322, Průhonice, Czech Republic; 4 Department of Experimental Plant Biology, Faculty of Science, Charles University, Viničná 5, Prague, 12844 Czech Republic; 5 Institute of Microbiology ASCR, Vídeňská, Praha, 1083, 142 20 Czech Republic; 6 Department of Microbiology, Viçosa Federal University (UFV), P. H. Rolfs Street, Viçosa, Minas Gerais CEP: 36570-900 Brazil; 7 Faculty of Science, University of South Bohemia, Branišovská, České Budějovice, 1760, 37005 Czech Republic; 8 Evolution, Biodiversité (ISYEB), Institut de Systématique, Muséum national d’Histoire naturelle, CNRS, Sorbonne Université, EPHE, 57 rue Cuvier, Paris, CP 39, F-75005 France

**Keywords:** Stoichiometry, Orchids, Trophic modes, Mycoheterotrophy, Nitrogen, Phosphorus

## Abstract

**Background:**

Mycorrhiza is a ubiquitous form of symbiosis based on the mutual, beneficial exchange of resources between roots of autotrophic (AT) plants and heterotrophic soil fungi throughout a complex network of fungal mycelium. Mycoheterotrophic (MH) and mixotrophic (MX) plants can parasitise this system, gaining all or some (respectively) required nutrients without known reciprocity to the fungus. We applied, for the first time, an ecological stoichiometry framework to test whether trophic mode of plants influences their elemental carbon (C), nitrogen (N), and phosphorus (P) composition and may provide clues about their biology and evolution within the framework of mycorrhizal network functioning.

**Results:**

We analysed C:N:P stoichiometry of 24 temperate orchid species and P concentration of 135 species from 45 plant families sampled throughout temperate and intertropical zones representing the three trophic modes (AT, MX and MH). Welch’s one-way ANOVA and PERMANOVA were used to compare mean nutrient values and their proportions among trophic modes, phylogeny, and climate zones. Nutrient concentration and stoichiometry significantly differentiate trophic modes in orchids. Mean foliar C:N:P stoichiometry showed a gradual increase of N and P concentration and a decrease of C: nutrients ratio along the trophic gradient AT < MX < MH, with surprisingly high P requirements of MH orchids. Although P concentration in orchids showed the trophy-dependent pattern regardless of climatic zone, P concentration was not a universal indicator of trophic modes, as shown by ericaceous MH and MX plants.

**Conclusion:**

The results imply that there are different evolutionary pathways of adaptation to mycoheterotrophic nutrient acquisition, and that the high nutrient requirements of MH orchids compared to MH plants from other families may represent a higher cost to the fungal partner and consequently lead to the high fungal specificity observed in MH orchids.

**Supplementary Information:**

The online version contains supplementary material available at 10.1186/s12870-023-04436-z.

## Background

Mycorrhiza is a ubiquitous form of symbiosis, since over 90% of plant species are estimated to form mutualistic relationships with soil fungi to access essential nutrients. In exchange for carbon (C) produced by plants in photosynthesis, soil fungi supply water and micro- and macroelements [1]. It is estimated that approximately 80% of nitrogen (N) and phosphorus (P), which are considered most limiting to plant growth [2–5], is provided by mycorrhizal fungi [6–8]. It has been frequently reported that hyphae of mycorrhizal fungi interconnect roots of various plants resulting in a complex web called Common Mycorrhizal Network (CMN), where nutrients and signalling compounds can be exchanged between connected plants [9, 10]. Functional evidence of such transfer is provided by mycoheterotrophic plants [11]. They are devoid of chlorophyll and derive all their nutrients, including organic C, from autotrophic plants via mycelium of shared mycorrhizal fungi and are therefore also called epiparasites [12–14]. In addition to MH plants, there are also green, photosynthetically active plants that may derive fungal C with little or no reciprocity to the mycorrhizal partner [15]. In this way they cover deficiencies of C resulting from low photosynthetic efficiency in the low-light habitats they occupy. Because they tap on two C sources, they are called mixotrophic or partially mycoheterotrophic [16–20].

Evaluation and quantification of resource translocation between plants sharing CMN are an ongoing challenge [21]. Although many experiments rely on stable isotopes to detect C and N nutrient flow within CMN, ecological stoichiometry (ES) also provides a useful framework linking differences in the composition of an organism’s multiple chemical elements and ecological interactions in an ecosystem to offer predictions about mycorrhizal functioning [22–25]. Indeed, the stoichiometry of the exchange of plant C for fungal P and N has been found to play a vital role in the rate and efficiency of resource exchange between mycorrhizal partners shaping not only the strengths of interactions, but also their mutualistic versus parasitic nature [22, 26]. Going further, N:K stoichiometry of soil as well as soil N and P concentrations have been identified as key determinants of the local occurrence and density of MH plants in tropical rainforest [27, 28]. A growing body of evidence also suggest that the rate and direction of resource transfer in CMNs reflect plant nutrient requirements [25, 29, 30]. This, within the ES framework, implies that both biochemical and physiological mechanisms of specialisation of MH plants to their fungal partner(s) could be reflected in the elemental composition of a plant.

Determining the elemental composition of MH plants may therefore help to understand the requirements of parasites of CMN for the main nutrients and ecology of this trophic mode, though such a determination has yet to be made.

All orchid species are MH during germination of their almost reserveless, dust-like seeds and subsequent early ontogenesis [12, 13, 31]. Only a minority of known orchid species stay fully MH in adulthood. Most of them develop green leaves when adult and become photosynthetic and autotrophic (AT) or mixotrophic (MX) if they continue to derive C from associated fungi while remaining photosynthetically active. Sometimes they produce variegated individuals [32], but rarely do MX orchids form achlorophyllous, non-photosynthetic variants called albinos, which survive fully on MH nutrition [17, 29].

Although most orchids are green, this family encompasses ca. 250 MH species and outnumbers other plant families in which this trophic mode has been identified [13, 33, 34]. In all, orchids are thus a good model to study the elemental composition of the organism in relation to trophic modes.

Interestingly, MH and MX orchids have been found to exhibit unusually high nitrogen concentration compared to autotrophic reference plants from the same habitats [14, 35–37]. Given the interdependence of N and P in biochemical functions and cellular components, N and P ratios are tightly linked [38], and we expect that MX and especially MH orchids are also enriched in P. Many terrestrial orchids, especially MH, have a simplified root system [39], which indicates a heavy reliance on mycorrhizal pathways for P and other nutrient uptake. Given that P plays a key role in the establishment and sustainability of plant-fungal symbioses [40–42] while also being a limiting factor of plant growth globally [5, 43, 44], high P and N requirement revealed by MH orchids may be a crucial factor shaping orchids’ ecological interactions. Functional diversity in plant–fungus combinations may lead to selection for fungi with required nutritional capability, as a driver of evolution of partner specificity and niche diversification [45, 46]. However, in contrast to N, the P concentrations in orchids as well as other plants showing various trophic modes remain largely unexplored [28, 47].

Here we introduce the first data on C:N:P stoichiometry for temperate orchids displaying three trophic modes, namely AT, MX and MH. We also present P concentration analyses extended to other plant groups with different trophic modes in both temperate and intertropical zones. Our questions are as follows: [1] Does elemental stoichiometry vary among orchids representing different trophic modes [2]? Do MX and MH stoichiometric C:N:P signatures reflect C shortage [3]? Is the tissue concentration of P influenced by trophic modes or is it family-dependent [4]? Can P concentration serve as an additional explanatory variable to characterise trophic modes?

## Materials and methods

### Sample collection and element measurements

Plant samples were collected and analysed in three sets. The first set (#1) comprised 200 samples collected between 2004 and 2020 from 10 localities covering both temperate and neotropical regions. The second set (#2) encompassed 602 samples collected from 13 populations of terrestrial plants growing in the European temperate zone harvested from 2017 to 2020. Taxonomic identification of the samples was carried out by the specialists listed in Additional file 1; Table S1A. Samples for which it was possible to make a voucher were deposited in Herbarium Universitetas Gedanensis – UGDA (Gdańsk, Poland), their numbers are given in the Additional file 1; Table S2. We used both sample sets to examine P content in a total of 802 samples representing 135 species and 45 families. Among them, orchids constituted 343 biological samples from 39 species, spanning the three trophic modes (AT, MX and MH). The third set (#3) mostly comprised orchid samples and a selection (26 samples) of reference autotrophic, non-orchid species (REF) from set #2. These samples were reanalysed for C and N concentrations to examine the C:N:P stoichiometry of three trophic modes in temperate orchids. Due to insufficient material for this analysis, each sample was a mix of two biological samples for a given species per given population (Additional file 1; Table S1B). In this set of 138 samples, AT orchids constituted 66% of samples, while MX and MH orchids accounted for 25% and 9%, respectively, which roughly reflects the proportion of species numbers in the orchid flora of temperate regions [48].

At each sampling site, leaves (or stem in the case of MH) were collected from 1 to 5 different individuals representing all trophic modes present locally, with autotrophic species serving as a reference. All plants were collected at the flowering stage, since P and N concentrations vary during a plant’s lifetime [49]. They were immediately placed in silica gel and, after transfer to the laboratory, oven-dried at 70 °C until constant weight and ground to a fine powder using a Quiagen TissueLyzer II.

Samples from set #1 were digested with nitric acid (HNO_3_, 65%), hydrogen peroxide (H_2_O_2_, 30%), and subsequently phosphate was analysed with a NIVO microplate reader (Perkin-Elmer France SAS, Villebon sur Yvette, France). The samples collected in Brazil and Japan were analysed in the country of origin. Perchloric acid was used for digestion of samples from set #2 and phosphate was analysed colorimetrically using flow-injection analysis (FIA Lachat QC8500, Lachat Instruments, USA). The P concentration data (set #1 and #2) were analysed together because the impact of the method on the variance was one hundred times smaller than the differences between the study groups. C and N percentages of plant tissue from set #3 were measured on a NC Elemental analyser (ThermoQuest, Germany). P concentration values for a given sample mix were obtained by averaging the results from the relevant samples from #2 P analysis. Units for leaf C, N and P were expressed as weight per gram of dry mass (g DM) and elemental ratio as molar units.

Species were assigned to AT, MX or MH trophic modes based on literature data on ^13^ C isotopic signature (enrichment in ^13^ C; Additional file 1; Table S3). There are some claims, based on ^18^O and ^2^ H isotopic signature [50], that some AT orchids with ^13^ C isotopic signature typical for autotrophs could be MX. Yet, these statements rely on evidence for a nutrient flow from the fungus to the orchid without data on the reverse flow: thus, there is no data on the direction of the net flow and thus no direct evidence for MX nutrition. We therefore restricted the MX category to the orchids and plants displaying characteristic ^13^ C isotopic signature, for which ability to extract fungal carbon is further supported by the existence of achlorophyllous variants [19]. Moreover, green plant species for which no stable isotope data were found in the literature were assumed AT, likely by large the most frequent status in orchids.

### Statistical analyses

For P concentration analysis (sets #1 and #2 pooled), samples were assigned to the following categories: trophy (AT, MX, MH), phylogeny (*Orchidaceae*, *Ericaceae*, B&G - *Burmaniaceae* and *Gentianaceae*) and climate (TROP - intertropical zone and TEMP - temperate latitudes; see, raw data in Additional file 3). The “climate” category acknowledges the well-known latitudinal change of N and P tissue concentration in plants [4]. In this study, the relation between P concentrations and latitudinal distribution of plant-sampling sites were described and tested by linear regression and Pearson correlation. Welch’s one-way ANOVA was used to compare mean nutrient values and their proportions among the categories studied due to unequal variances between groups, followed by the Games-Howell post-hoc test. All statistical analyses were performed in R [51] and based on logarithmically transformed, *x*’= log10(x) P data.

For C:N:P stoichiometry (set #3) samples were analysed as one factor: trophy with three categories represented by orchids (AT, MX and MH); autotrophic plants from various families were a reference (REF). To illustrate the level of multivariate similarity between the studied categories, non-metric multidimensional scaling (n-MDS) was performed based on the matrix of Euclidean distances between the samples. Prior to n-MDS, all values of C:N:P were standardised by variable and logarithmically transformed, *x*’= log(x + 1). Based on the same distance matrix, the differences between groups were tested by one-way PERMANOVA with *post-hoc* tests (999 permutations).

The growth rate hypothesis (GRH) posits that organisms with higher growth rates require disproportionately higher P than N concentrations, resulting in a scaling exponent of N to P concentrations below unity (i.e. α < 1.0) [52]. We conducted reduced major axis (SMA) regression to study the pattern of the leaf N versus P scaling relationship in relation to trophic modes AT and MH in orchids and reference AT plants (REF) for comparison. To perform the analyses, N and P concentration data from set #3 were logarithmically transformed, *x*’= log10(x). Analyses were performed in R package SMAtr 3 [53].

## Results

### Patterns of leaf C, N, P, and their stoichiometric ratios according to trophic modes

**Fig. 1:**
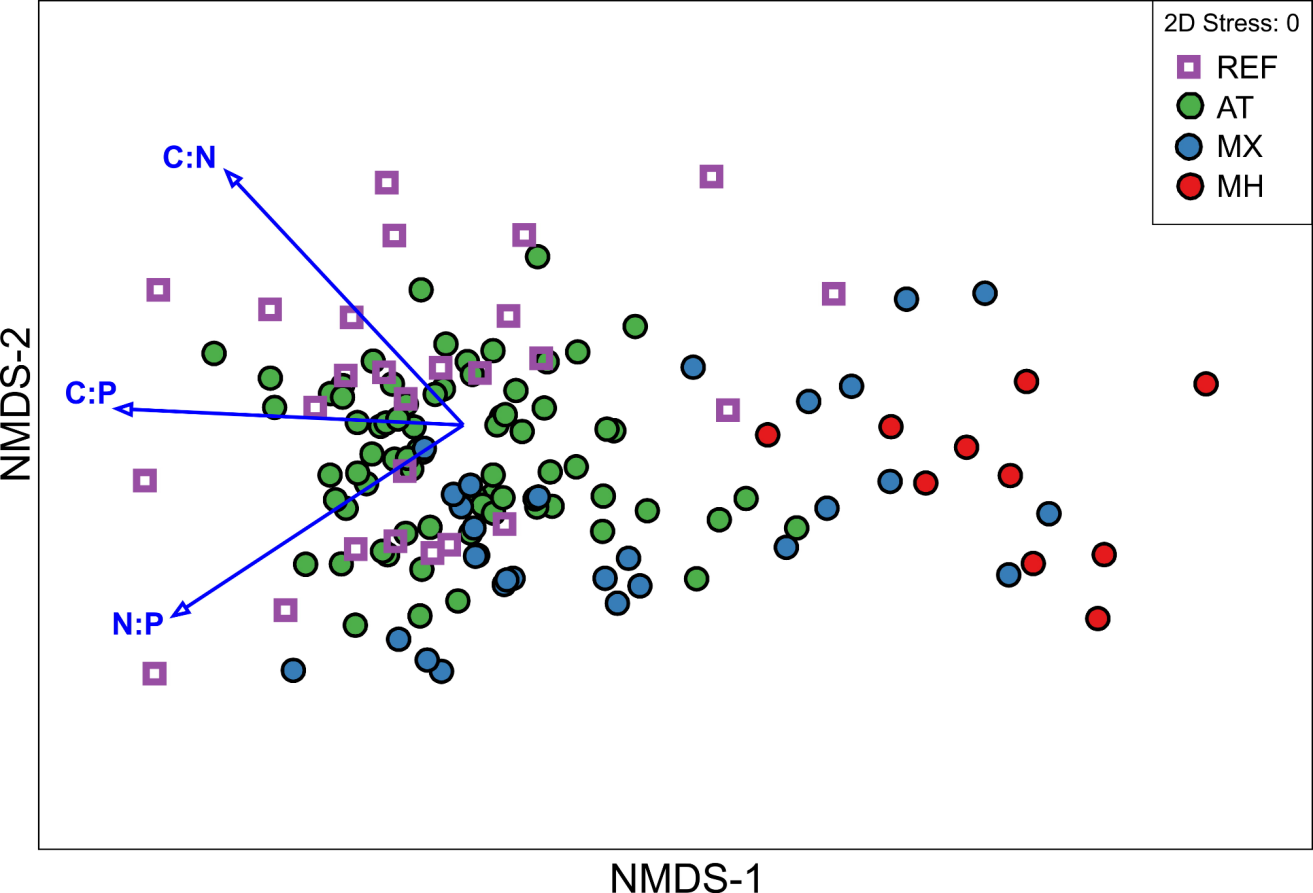
Non-metric multidimensional scaling (NMDS) plot showing differences in stoichiometric proportion of elements between tropic modes in orchids. Carbon (C), nitrogen (N), phosphorus (P) in orchid species representing different trophies: autotrophy (AT), mixotrophy (MX) and mycoheterotrophy (MH) in comparison with reference autotrophic non-orchid species (REF). Fitted vectors display the response variables: stoichiometric proportion of elements (molar) C:N, C:P and N:P in the ordination space and show the differences between the groups in association with these variables. (PERMANOVA: Pseudo-F = 36.189; P = 0.001, *p*-values based on 999 permutations)

**Fig. 2:**
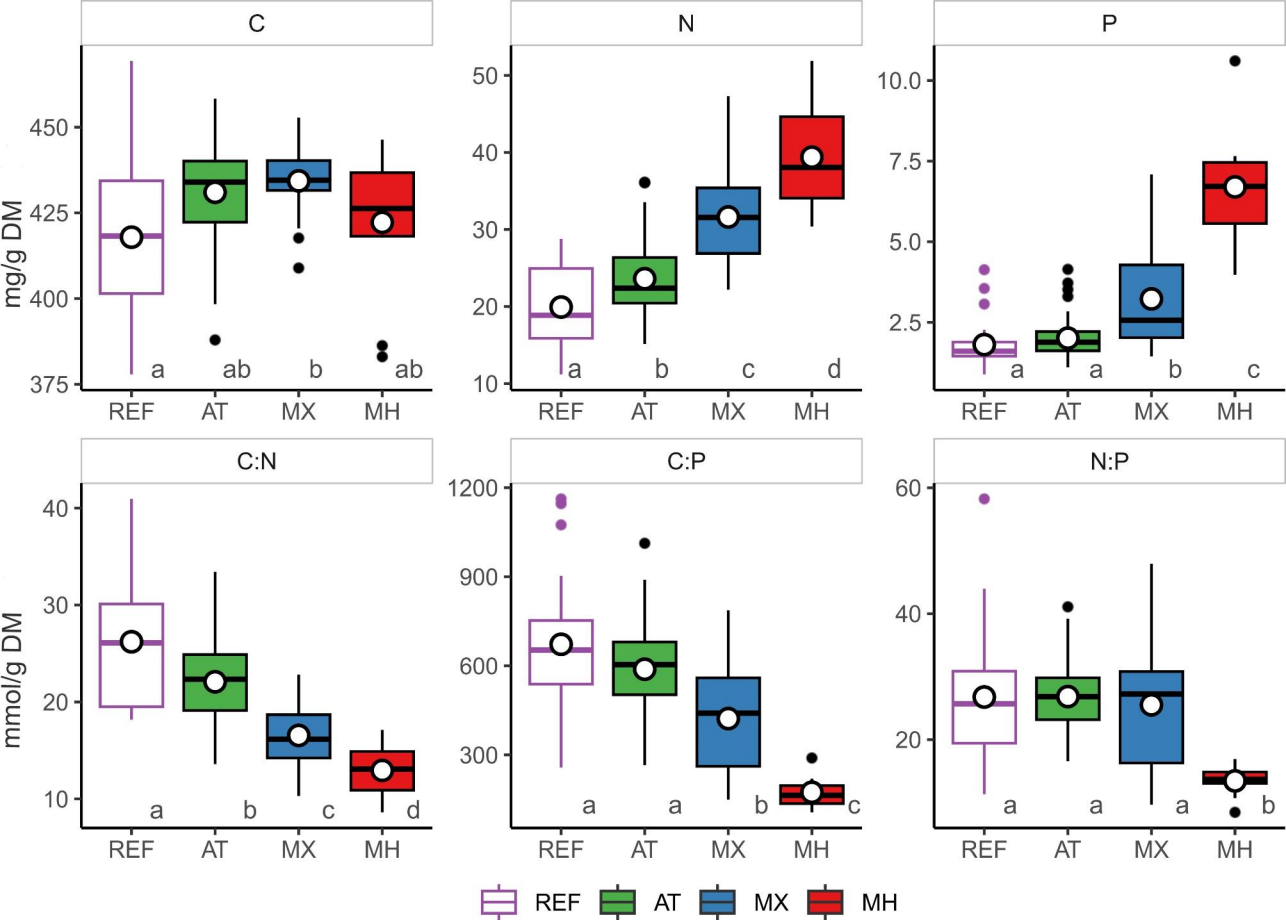
Concentration and molar ratios of elements in groups of orchids representing different tropic modes. average (horizontal bar), quartiles (box) and value range (whiskers) for carbon (C), nitrogen (N), phosphorus (P) concentration, and ratio of the elements, autotrophy (AT), mixotrophy (MX), mycoheterotrophy (MH), and reference autotrophic non-orchid species (REF). The letters above the bottom line indicate the significant differences at p < 0.05 based on the *post-hoc* Games-Howell test after Welch ANOVA showed significant differences between groups for both nutrient concentration and their proportions ( C - F = 4,17, df = 3, *p* = 0.01; .N - *F* = 42.9, df = 3, *p* < 0.001; P - *F* = 67.3, df = 3, *p* < 0.001; C:N - *F* = 35.8, df 3; *p* < 0.001; C:P - *F* = 58.3, df = 3, *p* < 0.001; N:P - *F* = 34.3, df = 3, *p* < 0.001). Values for C, N and P concentration are given in mg/g DM

Based on the results of the stoichiometric data pool, the average C:N:P molar ratio for AT, MX and MH orchids was 552:26:1, 503:31:1, and 220:14:1, respectively (see the raw data in Additional file 3). The trophy factor explained 44.8% of variation in the dataset (Fig. 1, Additional file 2; Table S4) and significantly (*p* = 0.001; Table 1) differentiated trophic groups based on their mean nutrient stoichiometric proportion: C:N (AT = 22.1, MX = 16.5, MH = 12.9 ), C:P (AT = 558, MX = 422, MH = 174), N:P (AT = 26.8, MX = 25.5, MH = 13.4) (Fig. 2, Additional file 2; Table S5). MH orchids significantly differentiated from AT, MX and REF with the lowest ratios of C:N and C:P and N:P (Fig. 2, Additional file 2; Table S5). The values of nutrient concentration and their stoichiometric ratios for MX trophic mode formed a continuum between AT and MH, except for N:P, which was similar in all green plants (AT, MX orchids and REF). In orchids, the highest variation coefficient (calculated as standard deviation / mean) of most parameters in MX further confirmed their intermediate position (Figs. 1 and 2, Additional file 2; Table S5).

**Table 1:** Results of a *post-hoc* pairwise test between groups of orchids representing different trophies: autotrophic (AT), mixotrophic (MX) mycoheterotrophic (MH) orchids and reference autotrophic non-orchid species (REF) with respect to stoichiometric ratios of nutrients: carbon, nitrogen and phosphorus. (PERMANOVA: Pseudo-F = 36.19; df = 3; p = 0.001; p -values based on 999 permutations)

Groups 1	Group 2	t	*p*
**AT**	**MX**	4.821	0.001
**AT**	**MH**	11.61	0.001
**AT**	**REF**	1.989	0.02
**MX**	**MH**	4.797	0.001
**MX**	**REF**	4.008	0.001
**MH**	**REF**	8.037	0.001

The mean elemental concentrations of orchid biomass significantly differed between trophic modes: N (AT = 23.7, MX = 31.6; MH = 39.4 mg N per g DM) and P (AT = 2.01, MX = 3.23; MH = 6.70 mg P per g DM), except for C (Fig. 2, Additional file 2; Table S5, S6). Hence, mean tissue concentration values for N and P increased markedly along the trophic gradient AT < MX < MH in orchids. The tendency also applied to a narrowed dataset including only the tribe *Neottieae* (Additional file 2; Table S5) and was also visible in the differences in average value of nutrient concentration in green MX (N = 27.4 mg/g DM, P = 3.20 mg/g DM) versus albino MH (N = 48.9 mg/g DM, P = 7.63 mg/g DM) variants of *Cephalanthera damasonium* (Mill.) Druce. It is noteworthy that temperate AT orchids have slightly but significantly higher mean N concentration (N = 23.6 mg per g DM; *p* = 0.019) compared to reference autotrophic non-orchid species (REF) (N = 19.9 mg/g DM). Thus, we may claim that terrestrial, temperate AT orchids tend to accumulate more N than AT non-orchids.

The scaling exponent of N to P concentrations dropped gradually from AT reference plants (α = 0.814) through AT orchids (α = 0.761) to reach the lowest value (α = 0.668) in MH orchids (Additional file 2; Fig. S1, Table S9). Although slopes of regression lines did not differ significantly between groups (likelihood ratio = 0.335; df = 2; p = 0.845), regression line shifts were significant (Wald = 116.1; df = 2; p < 0.001).

### Phosphorus concentration differences through trophy and phylogeny

**Fig. 3:**
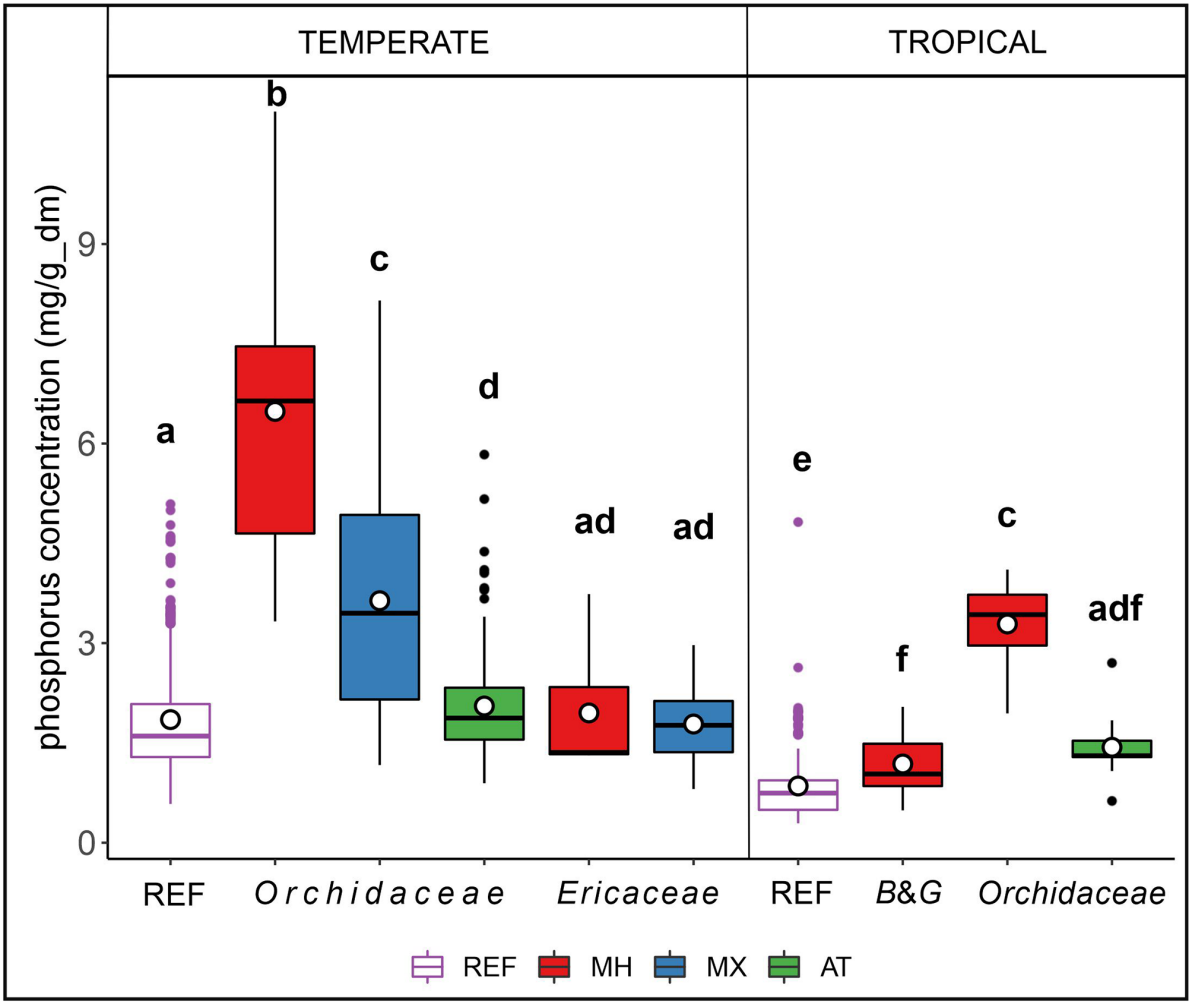
Concentration of phosphorus (P) in groups of species representing different biogeography, phylogenetic groups, and trophic modes. median (circle), average (horizontal bar), quartiles (box) and value range (whiskers); autotrophy (AT), mixotrophy (MX) and mycoheterotrophy (MH) and respective reference autotrophic non-orchid species (REF). Orchids include AT, MX and MH species; *Ericaceae* include MX and MH species, and B&G (*Burmaniaceae* and *Gentianaceae*) includes MH species only in our species set. The letters above the bottom line show the significant differences at p < 0.05 based on the *post-hoc* Games-Howell test after Welch ANOVA showed significant differences between groups (F = 178,9; df = 123,7; *p* < 0.001)

Analyses of pooled data from sets #1 and #2 demonstrated that P concentration in orchids increases along the trophic gradient AT < MX < MH and this trend stays salient irrespective of geographical range, as shown by significant differences between trophic modes in terrestrial orchids both in intertropical and temperate zones (Fig. 3, Additional file 2; Fig S2, Table S7). A significant relation between P concentrations and latitudinal distribution of plant-sampling sites (*p* < 0.001; Additional file 2; Fig. S2) revealed that P concentration increased with latitude. This tendency was probably the reason why the concentration of P in tropical MH orchids was significantly lower than in temperate ones (*p* < 0.001; Fig. 3, Additional file 2; Table S7).

AT terrestrial orchids from both temperate and tropical zones showed slightly but significantly higher P concentrations (*p* = 0.001) compared to reference AT non-orchid species (REF, Fig. 3, Additional file 2; Fig. S2, Table S7). A similar trend was not detected in set #3 (*p* = 0.28; Additional file 2; Table S6), which may be the result of a smaller data set than sets #1 and #2 pooled (Fig. 3, Additional file 2; Table S7). Thus, we may claim that terrestrial AT orchids regardless of climatic zone tended to accumulate more P than non-orchid autotrophic plants. Significantly higher P content compared to the AT reference (*p* < 0.001) is also a feature of tropical, non-orchid MH represented by *Gentianaceae* and *Burmaniaceae* (Fig. 3; B&G), which, unlike orchids, are associated with arbuscular mycorrhizal fungi. In contrast, temperate *Ericaceae*, which like orchids are associated with asco- and basidiomycetes, showed no differences, either in comparison with REF plants (*p* > 0.999) or between MX and MH within the family (*p* = 0.999) (Fig. 3, Additional file 2; Table S7).

## Discussion

### Nutrient concentration and stoichiometry vary between trophic modes in orchids

In this study, we provide for the first time the C:N:P stoichiometry of temperate orchids representing different trophic modes (AT, MX and MH). Various studies have confirmed that an increasing reliance on MH nutrition entails an increase in isotopic ^15^ N enrichment [37] and leaf N concentration in orchids [17, 29, 35, 36, 54]. Not only did we confirm the trend of growing N concentration along the trophic gradient (AT < MX < MH), but we also found the same trend for P. The analyses indicated that the mean N (39.4 mg/g DM) and P (6.70 mg/g DM) concentrations in leaves of MH orchids were nearly two (for N) and three times (for P) higher than in AT orchids and REF. Comparison of the respective values with the global average for temperate zone herbs (N = 22.4 mg g^− 1^ and P *=* 1.74 mg g^− 1^; [52]) also confirms the tendency of temperate MH orchids to have high nutrient accumulation. Remarkably, even AT orchids showed higher N and P concentrations than AT reference plants. One could wonder whether this may result from an admixture of truly AT orchids with hidden MX species. This is especially relevant, since some orchids associated with ‘rhizoctonias’ and usually considered AT are now claimed to receive organic matter from the ‘rhizoctonias’ fungi, based on their ^18^O and ^2^H isotopic signature [50]: yet the reverse flow from orchid to fungus remains unevaluated, so that the MX status of such orchids remains speculative. To exclude the possibility of hidden MX species into account, we analysed the frequency distribution of N (and P) concentration values in the AT orchid group: bimodality is expected if this group is not homogenous, yet a single mode was observed (Additional file 2; Fig. S3). In addition, the low (lowest of all tested groups) standard deviation of N and P concentrations among AT orchids further supports the claim of homogeneity (Additional file 2; Table S5). We rather believe that the extreme nutrient contents reflect a physiological particularity of this family, very possibly connected with their mycorrhizal fungi - the ‘rhizoctonias’, which are not known to be mycorrhizal in other plants [55]. The relatively large genome size typical of *Orchidaceae* [56] may also contribute to the higher average N and P levels in AT orchids compared to AT reference plants, as nucleic acids are rich in these elements. Although this hypothesis needs to be thoroughly tested, it is unlikely to affect calculations between trophic groups within the *Orchidaceae* as the average genome size is subfamily-specific [56–58] rather than trophic mode-specific (the largest genome in this study is represented by AT orchid - *Cypripedium calceolus* L.; see Additional file 2; Table S8).

The MX group of species formed an almost continuous gradient of nutrient concentration between AT and MH orchids, confirming its flexible mechanism of nutrient acquisition and use [18, 19, 59–61]. Surprisingly, Hynson et al. [37] showed no significant differences between MX and MH with respect to N concentration: we believe that this was due to the combination of temperate MH orchid species and the (sub)tropical ones, since the latter may have lowered the mean N concentration value due to the common feature of decreasing plant N concentrations towards the equator [4].

The phylogenetic signal connected with genetically and physiologically determined growth form, size and structure may have a major impact on plant stoichiometry [4, 62, 63]. However, we planned our studies to minimise bias by sampling within closely related species, applying a uniform sampling protocol and collecting plants at the same phenological stage to control for seasonal variation in foliar nutrient concentration [64]. We therefore expect the influence of the phylogenetic signal on the data to be negligible. The fact that all MX and MH samples (and 16% of AT samples) belong to the subfamily *Epidendroideae* indicates that, at least at the subfamily level, trophic modes, rather than phylogeny, predicted stoichiometry and N and P concentrations (Additional file 2; Table S5). This, in addition to the significant differences in P concentration between AT and MH orchids in the tropics, suggests a general trend in orchids, irrespective of climate zone and phylogenetic traits.

A possible explanation of the high N concentration in MX and MH orchids is acquisition of C in the form of amino acids [54, 65–67]. However, the reason for high P levels in orchids is unclear. At least the inorganic form of P can be transferred by fungi to AT orchids [47, 68]. Lysed fungal hyphae, which are on average richer in N and P than green plant tissue [69, 70], may also be the source of these nutrients [71, 72]. Indeed, differences in N concentration of fungal hyphae were found to influence N gain of MX *Epipactis* species [73]. However, digestion of the fungal matter inside root cells is commonly observed in many non-orchid MH [39] and AT plants [74], leaving an open question about the significance of this process in gaining exceptionally high P concentrations in orchids. However, it is a fact that the value of P concentration per unit of N concentration increases faster in MH orchids than in AT plants, including orchids, indicating differences in nutrient acquisition and/or recycling.

It is presumed that in the face of C deficiency, MH orchids reprogramme their metabolism to use N for energy [54, 75]. This raises the question of whether high P concentration shapes metabolism in a different way compared to AT plants or simply enlarges the P storage pool. Studies determining the contribution of the storage (inorganic P), structural (e.g., phospholipids) and biochemically functional (RNA, ATP) forms of P in MH orchids would clarify the functional significance of their high P concentrations. However, the lowest value of both scaling exponents of N to P and N:P coefficient of variation observed in MH orchids may point to high nutrient demand connected with high growth rate [23], which was also suggested based on transcriptome analyses [75].

Higher concentrations of P and N in orchids than in non-orchid MH plants [37] implying high nutrient demand for fungal community and CMN also raise the question of their implications for the evolution of orchid fungal specificity, demography and distribution. More studies showing the patterns of MH orchid demography in relation to the abundance and distribution of mycorrhizal fungi, taking into account environmental variables, as in [76], but also considering the stoichiometry of the soil and organisms (plants and fungi) supporting MH growth, could help to better understand the functioning MH orchids within CMN.

### Mycoheterotrophic lifestyle affects orchid stoichiometry

The low stoichiometric ratios (C:N, C:P and N:P) characteristic of MH orchids may result from passive reflection of those of their mycorrhizal fungi, as has been observed in fungi parasitising algae [77]. However, the higher mean (molar) C:N (14.4) and C:P (348) values in ectomycorrhizal fungi [69], from which partners of temperate MH orchids are recruited, may suggest the presence of a stoichiometric imbalance in the transfer pathway. To compensate for imbalance, consumers recycle C and nutrients differently using multiple mechanisms, including excretion of elements that are in excess [78]. In MH orchids, the demand for C met by uptake of N-rich sources of C [54] may result in an excess of N. Ammonium ions released at the orchid-fungus symbiotic interface as described in [66, 79] may simply be the result of excretion of excess N. Whether this is a sign of mutualism, as suggested by [65] or a way to mask “cheating” is debatable. Nevertheless, the actual existence of a stoichiometric imbalance in relation to C:N:P would have to be checked directly on the orchid-fungus pair.

While the concentration of C does not differ significantly between AT and MH orchids, various MH features suggest parsimonious C management: the typically small size of MH plants, their leaflessness and the short life-span of the stem [12], which limit the need for C-costly structural investment to build mechanically resistant cell walls [80]. Carbon limitation may be the main reason for extremely slow growth (up to several years) in the early ontogenesis phase when all orchids show MH nutrition [20]. Therefore, the nutritional composition of the adult MH plant, i.e. high P concentration and low (< 10) N:P ratio, as well as the low scaling component that usually is the hallmark of a fast-growing organism [23, 52, 81] may seem paradoxical. At least two possibilities can be considered here: [1] C, rather than N and/or P, is the main growth limiting factor [12]; [2] C is not the limiting factor during the flowering stage, e.g. due to the release of C stored in the rhizome or roots, and the flowering shoot of MH plants is indeed a fast-growing organ. In *Epipogium aphllum* Sw. the flowering shoot grows 4 cm per day and the plant completes its life cycle in a fortnight [82]. Presumed fast growth rate during flowering shoot expansion may imply a high nutrient demand, which may make the MH plant a strong physiological sink in mycorrhizal networks [83–85] further leading to high nutrient concentration. Although other physiological mechanisms associated with nutrient mass flow, such as high respiration demand of heterotrophic stem [75] or high transpiration rate [29] may contribute to the effect. More analyses of the vegetative parts of MH plants, which are slowly growing and may have less pronounced N and P demands, may test the second hypothesis. Either way, the requirements of the plant rather than fungal species have a stronger influence on resource flow, as exemplified by the different N concentrations of albino (MH) and green (MX) variants of *C. damasonium* sharing mycorrhizal fungi [29]. Higher N and P concentrations in the albino than in the green variant of *C. damasonium* found in our data also confirm this pattern.

### Phosphorus level is driven by trophic mode and phylogeny

Extended P concentration data across phylogeny, trophic modes, and climatic zones from sets #1 and #2 confirmed that P concentration in leaves of terrestrial orchids increases along the AT < MX < MH trophic gradient. This trend applies to all climatic zones, as despite generally lower P concentrations in tropical MH orchids than temperate ones, they show the same 3-fold higher P concentration relative to non-orchid reference AT plants. The lower P concentration in tropical MH orchids compared to temperate ones may simply be due to differences in P availability, which is lower in old, rain-washed tropical soils [5, 86]. This is supported by the same decreasing trend of N:P ratio with latitude observed in fungi [69].

However, P concentration is not a universal indicator of MH plant dependency on fungal resources, as exemplified by the lack of differences between ericaceous MH, MX and reference AT plants as well as high differences between *Burmaniaceae, Gentianaceae* and *Orchidaceae*. These findings may indicate different ecophysiological adaptation for nutrient acquisition from the fungi, developed during evolution on the path to mycoheterotrophy. Interestingly, it has already been shown that orchid and ericaceous MH species exhibit different enrichments in the stable isotopes of C and N as well as N concentration [37].

## Conclusions

Trophic modes in orchids i.e. autotrophy (AT) mixotrophy (MX) and mycoheterotrophy (MH), shape both the demand for C and nutrients (N and P) and their stoichiometry. A markedly increasing demand for N along the trophic gradient in orchids agrees with the data on the acquisition of N-rich C substrates. High P concentration relative to AT plants is an inherent feature of orchids regardless of climatic zone but does not characterise MH species from other families, indicating different, family-dependent factors shaping adaptation to mycoheterotrophic nutrient acquisition. Future studies should address the relationship between MH plant partner specificity and plant nutrient demand and the stoichiometric balance of resource flow between fungi and MH plants.

### Electronic supplementary material

Below is the link to the electronic supplementary material.

Supplementary Material 1

Supplementary Material 2

Supplementary Material 3

## Data Availability

The datasets supporting the conclusions of this article are included within the article and its additional files.

## Declarations

All methods were performed in accordance with the relevant guidelines and regulations.
