# Supplementary material for: Stoichiometry of carbon, nitrogen and phosphorus is closely linked to trophic modes in orchids
Source: BMC Plant Biol. 2023 Sep 12;23:422. doi: 10.1186/s12870-023-04436-z (PMC10496321; doi:10.1186/s12870-023-04436-z)
Supplement: Supplementary file 2 — Supplementary Material 2 [file 12870_2023_4436_MOESM2_ESM.docx]

**Stoichiometry of Carbon, Nitrogen and Phosphorus is Closely Linked with Trophic Modes in Orchids**

**Minasiewicz J., Zwolicki A., Figura T., Novotná A., Jersáková J., Bocayuva M.F, Selosse M.-A.**

**ADDITIONAL INFORMATION**

[**Table S4.** Results of PERMANOVA analysis testing differences between groups of orchids representing different trophies: autotrophic, mixotrophic, mycoheterotrophic and reference autotrophic non-orchid species with respect to stoichiometric ratios of nutrients: carbon, nitrogen and phosphorus. 2](#_Toc125668678)

[**Table S5.** Summary statistics of dataset #3 for carbon (C), nitrogen (N) and phosphorus (P) concentration and their proportions in orchid species representing different trophic modes: autotrophy (AT), mixotrophy (MX) and mycoheterotrophy (MH) in comparison with reference autotrophic plants (REF). ). x̄ – mean; s.d – standard deviation; c.v – coefficient of variation. All orchids from the MX and MH group belong to the subfamily Epidendroideae. 2](#_Toc125668679)

[**Table S6.** Results of post-hoc test Games-Howell test after Welch ANOVA for differences in carbon (C), nitrogen (N) and phosphorus (P) concentration and their proportions in orchid species representing different trophic modes: autotrophy (AT), mixotrophy (MX) and mycoheterotrophy (MH) in comparison with reference autotrophic plants (REF). 3](#_Toc125668680)

[**Table S7.** Results of Post-hoc Games-Howell test for Welch ANOVA (F = 178.9, d.f = 127.3, P < 0.001) testing differences in phosphorus (P) concentration between “zone.lf.t.s” factor categories. 4](#_Toc125668681)

[**Table S8.** Genome size (2C) of studied orchids along with their taxonomic placement within family Orchidaceae, trophic modes: autotrophy (AT), mixotrophy (MX) and mycoheterotrophy (MH). The non-parametric U Mann-Whitney test was performed to test differences between AT and MX groups only because the MH group was too small. The result of the test (U = 18; p = 0.126) shows a lack of statistically significant difference in the genome size between AT and MX orchids. 6](#_Toc125668682)

**Table S9**. Results of standardized major axis (SMA) regression between log10 values of leaf N and leaf P concentrations among groups of autotrophic orchids (AT), mycoheterotrophic orchids (MH) and non-orchids autotrophic reference plants (REF) along with slope and shift comparison. ……………………………………………….…….. 7

[**Fig. S1**. Relationships between leaf nitrogen and leaf phosphorus concentrations in terrestrial plants among groups of autotrophic orchids (AT), mycoheterotrophic orchids (MH) and non-orchids autotrophic reference plants (REF). Scaling exponents (α) were calculated from the standardized major axis (SMA) regression between log10 values of leaf N and leaf P concentrations. Resulted statistics of SMA and slope and shift comparison is given in the table below. 7](#_Toc141813966)

[**Fig. S2**. The effect of latitude on phosphorus (P) concentration. As latitude increases, P levels increase significantly in all plants representing different trophies, namely autotrophy (AT), mixotrophy (MX) and mycoheterotrophy (MH). The linear regression equation and Pearson correlation value and significance are given above the graph. 8](#_Toc141813967)

[**Fig. S3** Frequency distribution of nitrogen and phosphorus concentration of autotrophic (AT) orchids group. The lack of bimodal distribution suggests homogeneity of the group. ………………………... 8](#_Toc141813965)

**Table S4.** Results of PERMANOVA analysis testing differences between groups of orchids representing different trophies: autotrophic, mixotrophic, mycoheterotrophic and reference autotrophic non-orchid species with respect to stoichiometric ratios of nutrients: carbon, nitrogen and phosphorus.

| Source | df | SS | MS | Pseudo-F | P(perm) | Unique perm |
| --- | --- | --- | --- | --- | --- | --- |
| t | 3 | 24.384 | 8.1278 | 36.189 | 0.001 | 999 |
| Residuals | 134 | 30.096 | 0.2246 |  |  |  |
| Total | 137 | 54,479 |  | | | |

**Table S5.** Summary statistics of dataset #3 for carbon (C), nitrogen (N) and phosphorus (P) concentration and their proportions in orchid species representing different trophic modes: autotrophy (AT), mixotrophy (MX) and mycoheterotrophy (MH) in comparison with reference autotrophic plants (REF). ). x̄ – mean; s.d – standard deviation; c.v – coefficient of variation. All orchids from the MX and MH group belong to the subfamily Epidendroideae.

|  | N [mg/g_DM] | C [mg/g_DM] | P [mg/g_DM] | C:N mmol/g DM | C:P  mmol/g DM | N:P  mmol/g DM |  |  |  |  |  |
| --- | --- | --- | --- | --- | --- | --- | --- | --- | --- | --- | --- |
|  | | | | | | | |  |  |  |  |
| AT | 23.61 | 431.0 | 2.012 | 22.08 | 588.5 | 26.84 | x̄ |  |  |  |  |
|  | 4.641 | 14.64 | 0.562 | 4.306 | 141.0 | 5.210 | s.d |  |  |  |  |
|  | 0.196 | 0.034 | 0.279 | 0.195 | 0.240 | 0.194 | c.v |  |  |  |  |
|  | | | | | | | |  |  |  |  |
| MX | 31.62 | 434.3 | 3.228 | 16.54 | 422.3 | 25.53 | x̄ |  |  |  |  |
|  | 5.759 | 9.181 | 1.559 | 3.002 | 170.6 | 9.675 | s.d |  |  |  |  |
|  | 0.182 | 0.021 | 0.483 | 0.181 | 0.404 | 0.379 | c.v |  |  |  |  |
|  | | | | | | | |  |  |  |  |
| MH | 39.39 | 422.2 | 6.701 | 12.93 | 174.0 | 13.44 | x̄ |  |  |  |  |
|  | 6.709 | 21.13 | 1.696 | 2.719 | 53.00 | 2.400 | s.d |  |  |  |  |
|  | 0.170 | 0.050 | 0.253 | 0.210 | 0.304 | 0.178 | c.v |  |  |  |  |
|  | | | | | | | |  |  |  |  |
| REF | 19.93 | 431.0 | 1.811 | 26.21 | 672.5 | 26.77 | x̄ |  |  |  |  |
|  | 5.580 | 14.64 | 0.749 | 6.854 | 225.5 | 10.34 | s.d |  |  |  |  |
|  | 0.280 | 0.034 | 0.413 | 0.261 | 0.335 | 0.386 | c.v |  |  |  |  |

**Table S6.** Results of post-hoc test Games-Howell test after Welch ANOVA for differences in carbon (C), nitrogen (N) and phosphorus (P) concentration and their proportions in orchid species representing different trophic modes: autotrophy (AT), mixotrophy (MX) and mycoheterotrophy (MH) in comparison with reference autotrophic plants (REF).

| element | group1 | group2 | estimate | conf.low | conf.high | p.adj |
| --- | --- | --- | --- | --- | --- | --- |
| C:N | REF | AT | -0.15839 | -0.30724 | -0.00953 | 0.033 |
| C:N | REF | MH | -0.69555 | -0.93726 | -0.45385 | 8.07E-07 |
| C:N | REF | MX | -0.44466 | -0.60861 | -0.2807 | 2.57E-08 |
| C:N | AT | MH | -0.53717 | -0.75784 | -0.31649 | 7.43E-05 |
| C:N | AT | MX | -0.28627 | -0.39819 | -0.17434 | 6.84E-08 |
| C:N | MH | MX | 0.250899 | 0.023574 | 0.478224 | 0.029 |
| C:P | REF | AT | -0.10617 | -0.31184 | 0.099502 | 0.512 |
| C:P | REF | MH | -1.33277 | -1.65567 | -1.00987 | 1.41E-09 |
| C:P | REF | MX | -0.50142 | -0.79988 | -0.20297 | 0.000259 |
| C:P | AT | MH | -1.2266 | -1.51667 | -0.93653 | 3.34E-07 |
| C:P | AT | MX | -0.39525 | -0.64555 | -0.14496 | 0.000844 |
| C:P | MH | MX | 0.831343 | 0.483157 | 1.17953 | 3.57E-06 |
| N:P | REF | AT | 0.052218 | -0.15894 | 0.263376 | 0.907 |
| N:P | REF | MH | -0.63721 | -0.90133 | -0.37309 | 1.63E-06 |
| N:P | REF | MX | -0.05677 | -0.34384 | 0.230302 | 0.953 |
| N:P | AT | MH | -0.68943 | -0.88878 | -0.49009 | 1.93E-06 |
| N:P | AT | MX | -0.10899 | -0.33083 | 0.112859 | 0.55 |
| N:P | MH | MX | 0.580444 | 0.308185 | 0.852703 | 1.14E-05 |
| N | REF | AT | 0.190287 | 0.025332 | 0.355241 | 0.019 |
| N | REF | MH | 0.706151 | 0.488258 | 0.924045 | 1.05E-08 |
| N | REF | MX | 0.484602 | 0.306859 | 0.662344 | 3.44E-08 |
| N | AT | MH | 0.515865 | 0.337612 | 0.694118 | 9.11E-06 |
| N | AT | MX | 0.294315 | 0.18529 | 0.40334 | 1.75E-08 |
| N | MH | MX | -0.22155 | -0.40873 | -0.03437 | 0.018 |
| C | REF | AT | 0.031898 | -0.00092 | 0.064719 | 0.059 |
| C | REF | MH | 0.010597 | -0.04752 | 0.068709 | 0.954 |
| C | REF | MX | 0.039946 | 0.007033 | 0.07286 | 0.013 |
| C | AT | MH | -0.0213 | -0.07485 | 0.032246 | 0.631 |
| C | AT | MX | 0.008048 | -0.00698 | 0.023074 | 0.499 |
| C | MH | MX | 0.02935 | -0.02423 | 0.082925 | 0.384 |
| P | REF | AT | 0.138068 | -0.06656 | 0.342694 | 0.281 |
| P | REF | MH | 1.343364 | 1.041064 | 1.645664 | 1.27E-10 |
| P | REF | MX | 0.54137 | 0.249396 | 0.833345 | 5.41E-05 |
| P | AT | MH | 1.205296 | 0.940187 | 1.470404 | 1.17E-07 |
| P | AT | MX | 0.403302 | 0.160767 | 0.645837 | 0.000438 |
| P | MH | MX | -0.80199 | -1.12747 | -0.47652 | 1.66E-06 |

**Table S7.** Results of Post-hoc Games-Howell test for Welch ANOVA (F = 178.9, d.f = 127.3, P < 0.001) testing differences in phosphorus (P) concentration between “zone.lf.t.s” factor categories.

Codes of the groups are explained in the table below.

| group1 | group2 | estimate | conf.low | conf.high | p.adj |
| --- | --- | --- | --- | --- | --- |
| TEMP.AT.NO | TEMP.AT.O | 0,083433 | 0,018845 | 0,14802 | 0,002 |
| TEMP.AT.NO | TEMP.MH.NO | 0,040555 | -0,19852 | 0,27963 | 1 |
| TEMP.AT.NO | TEMP.MH.O | 0,972745 | 0,828392 | 1,117097 | 0 |
| TEMP.AT.NO | TEMP.MX.NO | -0,01594 | -0,16527 | 0,133393 | 1 |
| TEMP.AT.NO | TEMP.MX.O | 0,469682 | 0,356461 | 0,582904 | 1,88E-14 |
| TEMP.AT.NO | TROP.AT.NO | -0,42591 | -0,50045 | -0,35138 | 0 |
| TEMP.AT.NO | TROP.AT.O | -0,13666 | -0,3555 | 0,082179 | 0,413 |
| TEMP.AT.NO | TROP.MH.NO | -0,24859 | -0,34322 | -0,15396 | 1,78E-10 |
| TEMP.AT.NO | TROP.MH.O | 0,436528 | 0,341925 | 0,531132 | 1,32E-11 |
| TEMP.AT.O | TEMP.MH.NO | -0,04288 | -0,28252 | 0,196765 | 1 |
| TEMP.AT.O | TEMP.MH.O | 0,889312 | 0,743577 | 1,035047 | 0 |
| TEMP.AT.O | TEMP.MX.NO | -0,09937 | -0,25002 | 0,051283 | 0,474 |
| TEMP.AT.O | TEMP.MX.O | 0,38625 | 0,271107 | 0,501392 | 6,24E-14 |
| TEMP.AT.O | TROP.AT.NO | -0,50935 | -0,58687 | -0,43182 | 5,08E-13 |
| TEMP.AT.O | TROP.AT.O | -0,22009 | -0,43936 | -0,00083 | 0,049 |
| TEMP.AT.O | TROP.MH.NO | -0,33202 | -0,42893 | -0,23512 | 9,06E-14 |
| TEMP.AT.O | TROP.MH.O | 0,353095 | 0,256302 | 0,449888 | 1,54E-11 |
| TEMP.MH.NO | TEMP.MH.O | 0,93219 | 0,669746 | 1,194633 | 4,67E-11 |
| TEMP.MH.NO | TEMP.MX.NO | -0,05649 | -0,32101 | 0,208025 | 0,999 |
| TEMP.MH.NO | TEMP.MX.O | 0,429127 | 0,177653 | 0,680601 | 0,000157 |
| TEMP.MH.NO | TROP.AT.NO | -0,46647 | -0,70788 | -0,22506 | 6,48E-05 |
| TEMP.MH.NO | TROP.AT.O | -0,17722 | -0,4719 | 0,117464 | 0,559 |
| TEMP.MH.NO | TROP.MH.NO | -0,28915 | -0,53493 | -0,04336 | 0,013 |
| TEMP.MH.NO | TROP.MH.O | 0,395973 | 0,150483 | 0,641464 | 0,000473 |
| TEMP.MH.O | TEMP.MX.NO | -0,98868 | -1,18236 | -0,795 | 0 |
| TEMP.MH.O | TEMP.MX.O | -0,50306 | -0,67364 | -0,33249 | 2,46E-10 |
| TEMP.MH.O | TROP.AT.NO | -1,39866 | -1,54854 | -1,24878 | 3,33E-13 |
| TEMP.MH.O | TROP.AT.O | -1,10941 | -1,35116 | -0,86765 | 1,20E-12 |
| TEMP.MH.O | TROP.MH.NO | -1,22134 | -1,38077 | -1,0619 | 0 |
| TEMP.MH.O | TROP.MH.O | -0,53622 | -0,69513 | -0,37731 | 2,04E-11 |
| TEMP.MX.NO | TEMP.MX.O | 0,485618 | 0,311019 | 0,660218 | 0 |
| TEMP.MX.NO | TROP.AT.NO | -0,40998 | -0,5646 | -0,25535 | 4,72E-10 |
| TEMP.MX.NO | TROP.AT.O | -0,12072 | -0,3647 | 0,123251 | 0,778 |
| TEMP.MX.NO | TROP.MH.NO | -0,23265 | -0,39647 | -0,06884 | 0,000691 |
| TEMP.MX.NO | TROP.MH.O | 0,452464 | 0,28917 | 0,615759 | 5,50E-11 |
| TEMP.MX.O | TROP.AT.NO | -0,8956 | -1,01637 | -0,77482 | 0 |
| TEMP.MX.O | TROP.AT.O | -0,60634 | -0,83651 | -0,37618 | 4,68E-07 |
| TEMP.MX.O | TROP.MH.NO | -0,71827 | -0,85157 | -0,58498 | 2,28E-14 |
| TEMP.MX.O | TROP.MH.O | -0,03315 | -0,16588 | 0,099573 | 0,998 |
| TROP.AT.NO | TROP.AT.O | 0,289253 | 0,068573 | 0,509933 | 0,007 |
| TROP.AT.NO | TROP.MH.NO | 0,177323 | 0,07383 | 0,280817 | 7,72E-06 |
| TROP.AT.NO | TROP.MH.O | 0,862442 | 0,759238 | 0,965646 | 3,19E-12 |
| TROP.AT.O | TROP.MH.NO | -0,11193 | -0,33657 | 0,112711 | 0,719 |
| TROP.AT.O | TROP.MH.O | 0,573189 | 0,34876 | 0,797618 | 2,85E-06 |
| TROP.MH.NO | TROP.MH.O | 0,685119 | 0,567575 | 0,802662 | 4,52E-10 |

Explanation of group code abbreviations used in the table S7

| Grup codes | Group description |
| --- | --- |
| TROP.MH.O | Tropical, MH  *Orchidaceae* |
| TROP.MH.NO | Tropical, MH *Burmaniaceae and Gentianaceae* |
| TROP.AT.O | Tropical, AT  *Orchidaceae* |
| TROP.AT.NO | Tropical, AT -Reference plant group |
| TEMP.AT.O | Temperate, AT *Orchidaceae* |
| TEMP.AT.NO | Temperate, AT Reference plant group |
| TEMP.MX.O | Temperate, MX *Orchidaceae* |
| TEMP.MX.NO | Temperate, MX *Ericaceae* |
| TEMP.MH.O | Temperate, MH *Orchidaceae* |
| TEMP.MH.NO | Temperate, MH *Ericaceae* |

**Table S8.** Genome size (2C) of studied orchids along with their taxonomic placement within family Orchidaceae, trophic modes: autotrophy (AT), mixotrophy (MX) and mycoheterotrophy (MH). The non-parametric U Mann-Whitney test was performed to test differences between AT and MX groups only because the MH group was too small. The result of the test (U = 18; p = 0.126) shows a lack of statistically significant difference in the genome size between AT and MX orchids.

| Orchid species | Subfamily | Trophy | Genome size (2C) |
| --- | --- | --- | --- |
| *Dactylorhiza fuchsii* | Orchidoideae | AT | 5.730^2^ |
| *Pseudoorchis albida* | Orchidoideae | AT | 9.338^3^ |
| *Orchis purpurea* | Orchidoideae | AT | 10.008^3^ |
| *Traunsteinera globosa* | Orchidoideae | AT | 12.346^3^ |
| *Platanthera chlorantha* | Orchidoideae | AT | 12.828^3^ |
| *Dactylorhiza majalis* | Orchidoideae | AT | 14.240^2^ |
| *Dactylorhiza sambucina* | Orchidoideae | AT | 15.804^1^ |
| *Gymnadenia conopsea* | Orchidoideae | AT | 16.132^1^ |
| *Orchis mascula* | Orchidoideae | AT | 19.726^1^ |
| *Epipactis palustris* | Epidendriodeae | AT | 23.069^3^ |
| *Orchis militaris* | Orchidoideae | AT | 24.147^1^ |
| *Platanthera bifolia* | Orchidoideae | AT | 27.830^1^ |
| *Neottia ovata* | Epidendriodeae | AT | 36.792^1^ |
| *Cypripedium calceolus* | Cypripedioideae | AT | 80.294^1^ |
| *Limodorum abortivum* | Epidendriodeae | MX | 10.751^3^ |
| *Epipactis atrorubens* | Epidendriodeae | MX | 27.961^1^ |
| *Epipactis helleborine* | Epidendriodeae | MX | 27.961^1^ |
| *Cephalanthera longifolia* | Epidendriodeae | MX | 36.430^1^ |
| *Cephalanthera damasonium* | Epidendriodeae | MX | 37.819^1^ |
| *Neottia nidus-avis* | Epidendriodeae | MH | 33.933^3^ |

^1^Rewers M, Jedrzejczyk I, Rewicz A, Jakubska-Busse A. Genome Size Diversity in Rare, Endangered, and Protected Orchids in Poland. *Genes*. 2021; 12(4):563. <https://doi.org/10.3390/genes12040563>

^2^ Eriksson M.C, Mandáková T, McCann J, Temsch EM, Chase MW, Hedrén M, Weiss-Schneeweiss H, Paun O. Repeat Dynamics across Timescales: A Perspective from Sibling Allotetraploid Marsh Orchids (*Dactylorhiza majalis* s.l.). Mol Biol Evol. 2022 Aug 3;39(8):msac167. doi: 10.1093/molbev/msac167. PMID: 35904928; PMCID: PMC9366187.

^3^ Smarda, P., Knápek, O., Šilerová, A., Horová, L.,Grulich, V., et al. (2019). Genome sizes and genomic guanine+cytosine (GC) contents of the Czech vascular flora with new estimates for 1700 species. Preslia. 91. 117-142. 10.23855/preslia.2019.117.014

**Fig. S**
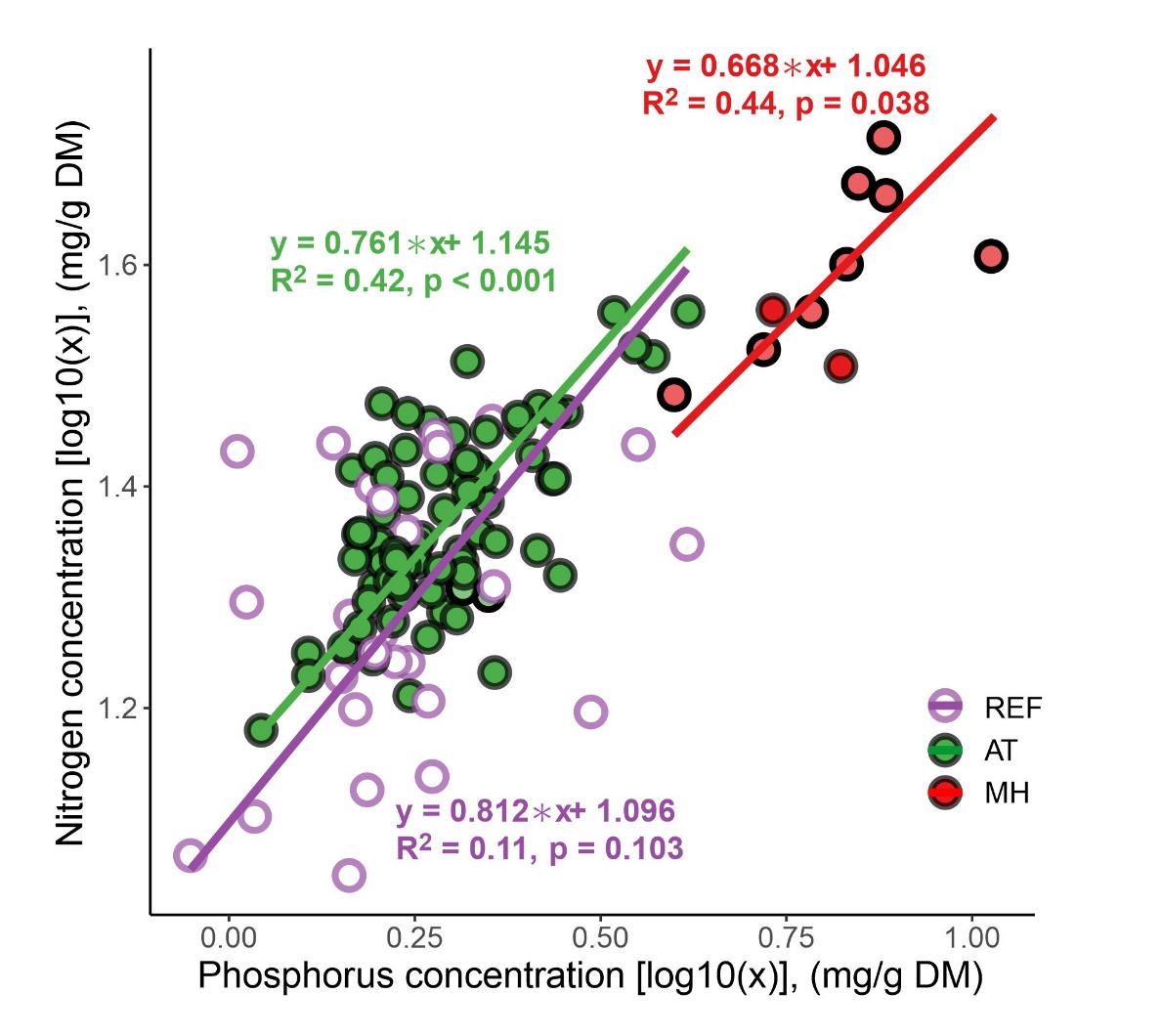
**1**. Relationships between leaf nitrogen and leaf phosphorus concentrations in terrestrial plants among groups of autotrophic orchids (AT), mycoheterotrophic orchids (MH) and non-orchids autotrophic reference plants (REF). Scaling exponents (α) were calculated from the standardized major axis (SMA) regression between log10 values of leaf N and leaf P concentrations.

**Table S9**. Results of standardized major axis (SMA) regression between log10 values of leaf N and leaf P concentrations among groups of autotrophic orchids (AT), mycoheterotrophic orchids (MH) and non-orchids autotrophic reference plants (REF) along with slope and shift comparison.

| **Group** | **n** | **Intercept** | **SMA slope (α)** | **95%CI** | **R2** | **P-value** | **Slopes comparison** | **Shift comparison** |
| --- | --- | --- | --- | --- | --- | --- | --- | --- |
| REF | 26 | 1,096 | 0,812 | 0.55-1.20 | 0,11 | 0,103 | Likelihood ratio = 0.335 | Wald = 116.1 |
| AT | 74 | 1,145 | 0,761 | 0.64-0.91 | 0,42 | < 0.001 | df= 2 | df= 2 |
| MH | 10 | 1,046 | 0,668 | 0.37-1.19 | 0,44 | 0,038 | P-value = 0.845 | P-value < 0.001 |

**
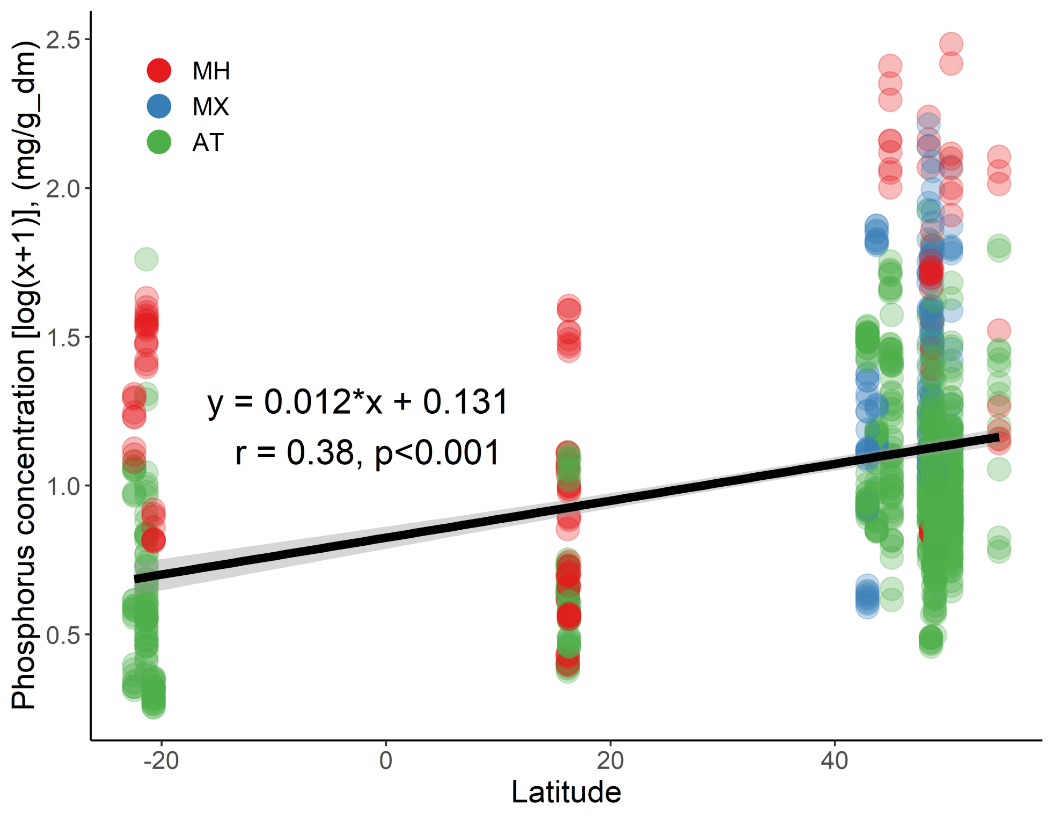
**

**Fig. S2**. The effect of latitude on phosphorus (P) concentration. As latitude increases, P levels increase significantly in all plants representing different trophies, namely autotrophy (AT), mixotrophy (MX) and mycoheterotrophy (MH). The linear regression equation and Pearson correlation value and significance are given above the graph.

**Fig. S3** Frequency distribution of nitrogen and phosphorus concentration of autotrophic (AT) orchids group. The lack of bimodal distribution suggests homogeneity of the group.
